# Do gifted children without specific learning disabilities read more efficiently than typically developing children?

**DOI:** 10.3389/fpsyg.2024.1436710

**Published:** 2024-09-26

**Authors:** Laurent Lesecq, Laurent Querne, Julie Gornes, Laura Buffo, Louise Corbel, Anne Gaelle Le Moing, Patrick Berquin, Béatrice Bourdin

**Affiliations:** ^1^Department of Pediatric Neurology and Referral Center for Language and Learning Disorders, Amiens-Picardie University Medical Center, Amiens, France; ^2^Psychology Research Center - Cognition, Psychism, Organizations, PRC-CPO (EA7273), Jules Verne University of Picardie, Amiens, France; ^3^Groupe de Recherches sur l’Analyse Multimodale de la Fonction Cérébrale (GRAMFC) INSERM U1105, Amiens, France; ^4^University Department of Education and Training in Speech Therapy, Paul Sabatier University, Toulouse, France

**Keywords:** gifted children, Reading abilities, specific learning disabilities, pathological threshold, Wechsler scales

## Abstract

**Introduction:**

There are no published data on the written language skills of gifted children (GC). The objective of the present study was to evaluate reading abilities of GC vs. normative data from typically developing French children (TDC). Like English, French is considered to be an opaque language.

**Method:**

GC completed the Wechsler Intelligence Scales and a battery of language tests. Only children with a score two standard deviations (SD) above the norm were included. GC with current or past academic difficulties or specific learning disorders were excluded. The GC’s scores were compared with TDC’s normative scores for language tests in a chi-square-test and corrected for multiple comparisons.

**Results:**

Forty-five GC were included. The highest GC’s mean scores were for the WISC’s Verbal Comprehension Index (VCI) and the lowest for the Processing Speed Index (from more than two SDs to one SD higher above the TDC’s normative scores). GC were between 1.3 and 4.7 times more likely than TDC to achieve a high score. After correction, the distributions of the GC’s and TDC’s scores differed significantly with regard to spoonerism, phoneme deletion, and rapid automatic naming (*p* < 0.001), word and sentence repetition (*p* ≤ 0.007), and the reading of meaningful text (*p* = 0.03). GC and TDC did not differ significantly for reading meaningless texts and spelling accuracy.

**Discussion:**

As described in the literature, the GC in the present study had heterogeneous scores on the Wechsler Intelligence Scales. The GC performed better than TDC in assessments of the underlying skills of reading and when reading of meaningful texts. This advantage was lost in the absence of context, as shown by the lack of significant GC vs. TDC differences for reading meaningless texts and for spelling accuracy. Hence, GC presented a heterogeneous profile with regard to the underlying skills of reading and reading abilities. The present data should help to improve our understanding of GC’s reading skills. In particular, it is now essential to determine which written language tests and which score thresholds are appropriate for identifying specific learning disorders in GC.

## Introduction

Over the years, numerous models and definitions of giftedness have been proposed (Worrell et al., 2019), but to date no consensus has been reached on the precise meaning of giftedness. However, intellectual giftedness is frequently identified by standardized measures of intelligence. According to the American Psychological Association’s Dictionary of Psychology (American Psychological Association, 2018), giftedness is defined as a Full-Scale Intelligence Quotient (FSIQ) of 130 or more (corresponding to two standard deviations (SDs) above the population average). Although the FSIQ are frequently used to identify intellectual giftedness, there is no consensus among experts on the best methods and criteria for identifying and assessing superior cognitive abilities (Hodges et al., 2018). For example, the use of single index vs. FSIQ is subject to debate in the literature (Pereira-Fradin et al., 2010; Liratni and Pry, 2007, 2012; Farmer et al., 2021; Watkins and Canivez, 2022). Furthermore, inter-indexes differences increase with intellectual performance and might mask the identification of some gifted children (GC; Labouret and Gregoire, 2018). The levels of performance for reading and writing are at least partly related to the level of intellectual ability. Specifically, research suggests that vocabulary skills (Stanovich, 2000; Gough and Tunmer, 1986; Gavard et al., 2023) and the knowledge of text structure (Duke and Cartwright, 2021) enhance the speed of reading.

Over the past two decades, scientific research has considerably enriched our understanding of how GC operate on the cognitive level. However, few studies have examined the language skills of GC. As noted in the recent review by Bucaille et al. (2022), GC have a higher lexical capacity than their typically developing peers. Similarly, there are few literature data on the reading skills of GC. Indeed, to the best of our knowledge, GC’s reading skills have not previously been comprehensively studied and a few studies have focused solely on the reading skills of dyslexic GCs (Kranz et al., 2024; Van Viersen et al., 2015, 2016). Most of these studies were conducted in English, even though it is known that the characteristics of a language have an important impact on reading skills. Indeed, the identification of written words in the reading process depends on the written language’s degree of opacity (degree of correspondence between the spelling and the phonology of the language). In their written forms, English and French are both considered to be opaque languages (Caravolas et al., 2019; Landerl et al., 2022; Paulesu et al., 2001). Learning to read in English appears to be more difficult than in other European languages (Seymour et al., 2003), and grapheme-phoneme decoding skills are less effective in English dyslexics than in German dyslexics, for example (Ziegler et al., 2003). Arffa (2007) found that only 28% of the variance in the reading scores of typically developing children (TDC) was explained by intelligence and emphasized the need to further investigate this complex relationship. Although the reading level is known to be related to intelligence, a lack of research on this topic means that there is a significant gap in our understanding of GC’s ability to read in English and French.

**Figure 1:**
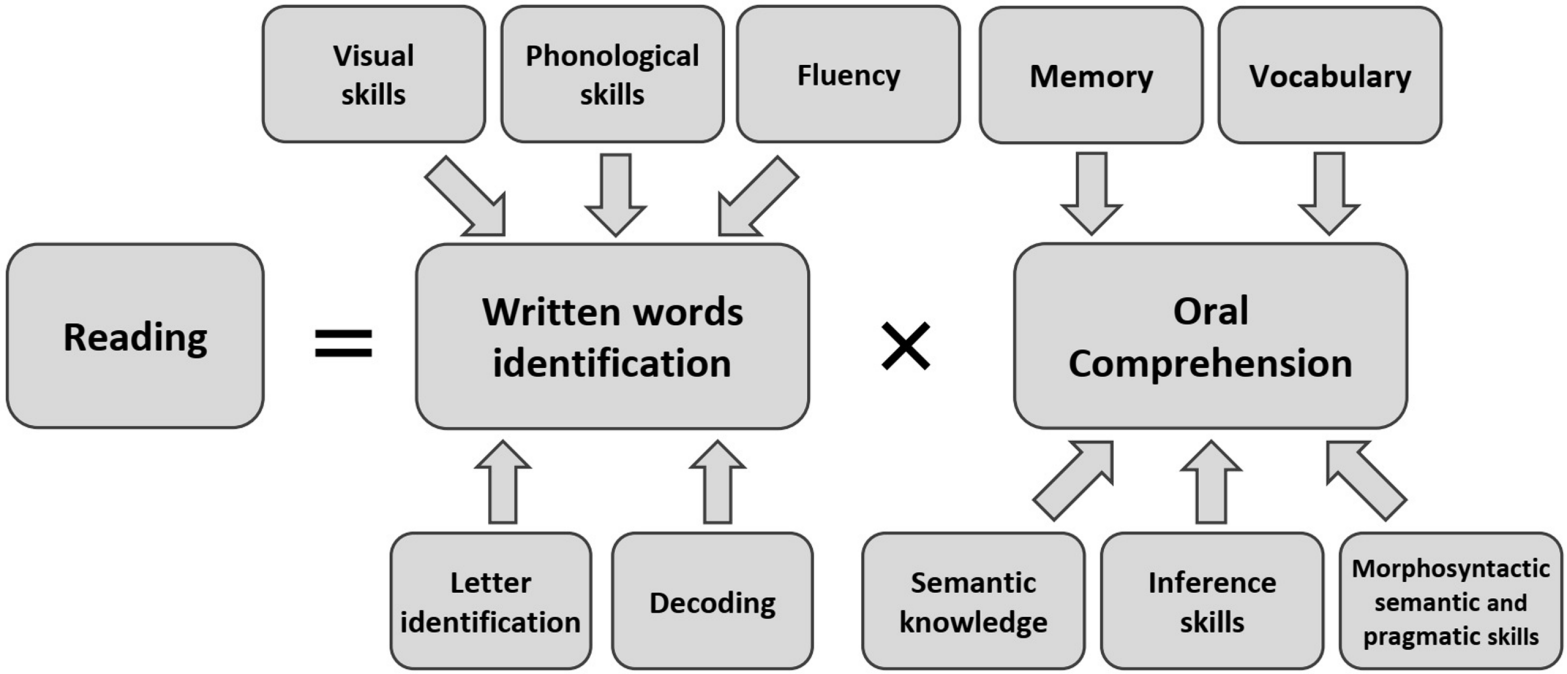
The Simple View of Reading (SVR) model by Gough and Tunmer (1986), revised by Sprenger and Ziegler (2019).

The Simple View of Reading (SVR) model (Gough and Tunmer, 1986) considers that reading ability (i.e., the ability to understand written language) has two fundamental components: written word recognition (i.e., decoding) and language comprehension (i.e., oral language skills). Sprenger-Charolles and Ziegler’s (2019) adaptation of the SVR model (see Figure 1) distinguishes between various cognitive skills involved in decoding and in listening comprehension.

As pointed by Kranz et al. (2024) in their recent review of reading and reading disorders in GC, there are no detailed literature data on reading skills. Kranz et al. pointed out that this type of data is essential for accurately diagnosing Specific Learning Disability (SLD) and precisely understanding the children’s reading profiles. The objective of the present study (conducted in France) was to investigate the reading skills and cognitive profiles of GC without learning disabilities. Lastly, we discuss our results with regard to the diagnosis of reading disorders in GC.

## Method

### Participants

Study participants were recruited through institutions offering educational programs for GC, associations for GC, and healthcare professionals. Each child and his/her legal guardian(s) received a study information sheet and gave their written, informed consent. The study was approved by the local investigational review board [*CPP Nord-Ouest II* (Amiens, France)]; reference: PI2021_843_0098. The study database was registered with the French National Data Protection Commission [*Commission nationale de l’informatique et des libertés* (Paris, France); reference: 2208336 v 0].

The children included in the study were recruited from specific classes for GC children in private schools. All the participating GC completed the Wechsler Intelligence Scale for Children, 4^th^ or 5^th^ edition (WISC-IV or V) or the Wechsler Preschool and Primary Scale of Intelligence, 4^th^ edition (WPPSI-IV). In line with the recommendations of Goldschmidt and Brasseur (2021) and Grégoire (2021), only children who scored 130 or more for at least one of the following reasoning indexes were included in the study: the Verbal Comprehension Index (VCI), the Visual Spatial Index (VSI), and the Fluid Reasoning Index (FRI) for the WISC-V and WPPSI-IV, and the VCI and the Perceptual Reasoning Index (PRI) for the WISC-IV. Children with ongoing or past academic difficulties, SLD, or psychiatric or neurological disorders were not included in the study.

### Material

The GC’s passive vocabulary and listening comprehension skills were evaluated using the French adaptation of the revised Peabody Picture Vocabulary Test [*Evaluation du Vocabulaire en Images Peabody* (EVIP); Dunn and Theriault-Whalen, 1993; Figure 1]. The children were asked to name one of four items that best corresponded to the word spoken by the examiner. Performance was reported as a standardized score (mean = 100, SD = 15).

The *EVAluation du Langage Ecrit et du langage Oral 6-15 ans* (EVALEO) is currently the most comprehensive, standardized, computerized battery for the assessment of written and spoken French language ability in children aged 6 to 15 (Launay et al., 2018a). Each year group corresponds to an average of 145 schoolchildren, ranging from *cours préparatoire* (Year 2/first grade) to *troisième* (Year 10/ninth grade). The distribution of EVALEO scores do not conform to a Gaussian distribution but range from S1 (abnormal) to S7 (very above-average) and correspond to the following percentile intervals: S.1 < 7%, S.2 [7–20%], S3 [21–38%], S.4 [39–62%], S.5 [63–80%], S.6 [81–93%], and S.7 > 93%.

The EVALEO tests were chosen to assess all the cognitive domains in the SVR model, in accordance with the guidelines for good practice in the assessment, prevention and remediation of written language disorders published by the French College of Speech Therapy (Leloup et al., 2022). The tests assessed various aspects of underlying reading skills such as spoonerism, phoneme deletion, and speed and accuracy of rapid color denomination (i.e., rapid automatic naming (RAN)). The tests also assessed the ability to read pseudowords, logatoms (i.e., meaningless words), and meaningless and meaningful texts, as well as spelling accuracy in a dictation. Although these markers cannot be used as individual diagnostic criteria for SLD, they are nonetheless integral elements of learning disability assessment protocols (Saksida et al., 2016; Colé and Sprenger-Charolles, 2021). Hence, they were included to provide standards for these skills within our sample. Furthermore, children with stronger cognitive reasoning abilities (particularly in the verbal domain) achieve higher scores in metaphonology tests than children with weaker cognitive reasoning abilities (McBride-Chang and Manis, 1996). Within the EVALEO population (around 1,500 children), males represent 47% of the sample, females 53%. Three types of pathology were taken into account in the EVALEO sample: dyslexia, dysorthographia and specific language impairment, and their possible combinations. Around 8% of children tested had one or more pathologies, including 6% of girls and 9% of boys. This figure of 8% corresponds to a percent commonly found in France. The distribution of socio-professional categories of parents of children from EVALEO is quite similar to the national statistics provided by the Institut National de la Statistique et Etudes Economiques (INSEE; Launay et al., 2018b) with the exception of an over-representation of the categories “manager, higher intellectual profession” and an under-representation of the “worker” category.

### Statistical analysis

Inter-index differences in the VCI, VSI, PRI, Working Memory Index (WMI) and Processing Speed Index (PSI) were tested with Student’s t-test for repeated measures or (if the latter could not be applied) Wilcoxon’s test.

The EVALEO test results were grouped together, as follows: S.1 with S.2 (S.12 ≤ 20%), S.3 with S.4 and S.5 (S.345 [21–80%]), and S.6 with S.7 (S.67 > 80%). Given that an average of 145 schoolchildren per school year completed the EVALEO battery, we considered that the S.12 group contained 29 TDC, S.345 contained 87 TDC, and S.67 contained 29 TDC children.

Firstly, we calculated the proportion ratios (*p*-ratios) [95% confidence interval (CI)] of GC vs. TDC for the EVALEO S.12 and S.67 scores in order to quantify the expected superiority of GCs over the TDCs of the EVALEO (EVALEO-TDC). Hence, the *p*-ratio for S.12 was defined as [n_(GC)_ with S.12/all_(GC)_]/[n_(EVALEO-TDC)_ with S.12/all_(EVALEO-TDC)_], and the *p*-ratio for S.67 was defined as [n_(GC)_ with S.67/all_(GC)_]/[n_(EVALEO-TDC)_ with S.67/all_(EVALEO-TDC)_].

Secondly, comparisons of proportions between GC vs. EVALEO-TDC for S.12, S.345 and S.67 were conducted with the chi-squared test.

The Benjamin-Holchberg test was used to check the alpha risk inflation for multiple comparisons in the WISC/WPPSI index (i.e., three GC intragroup comparisons) and the S.12, S.345 and S.67 *p*-ratios (i.e., 12 GC/EVALEO-TDC intergroup comparisons). All the *p*-values below are reported after Benjamini-Holchberg correction (statistical-tests: k = 15; significance: *p* < 0.05).

## Results

Forty-seven children (age range: 8 to 15) were initially included in the study. Two of the 47 were excluded due to low reading scores; hence, 45 children [34 boys (75.6%) and 11 girls (24.4%)] were included in the analysis.

### Intellectual and passive vocabulary efficiency

**Figure 2:**
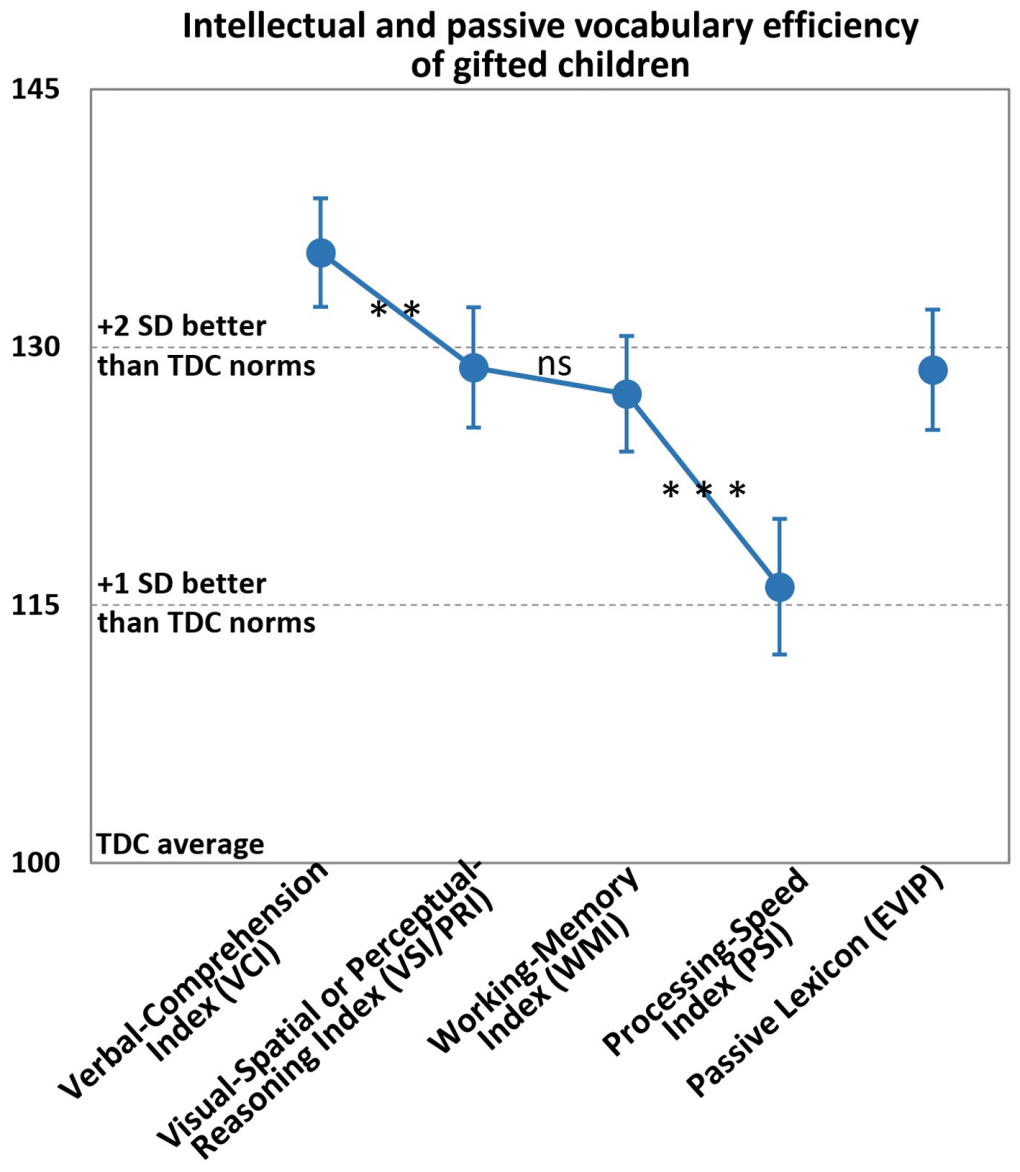
Mean scores obtained by GC to the WISC or WPPSI and EVIP. Vertical bars represented the 95%-CI. The *p*-values were given after Benjamini-Hochberg corrections for multiple comparisons with k = 15 (***, *p* < 0.001; **, *p* < 0.01; *, *p* < 0.05). SD: standard deviation; TDC, typically developmental children; ns, non significant.

Regarding intellectual efficiency, the mean scores of GC were more than two SDs higher than the TDC norms for the VCI (135.5, 95%-CI [132.4–138.7]). Compared with the VCI, the scores were progressively lower for the VSI/PRI (128.8, 95%-CI [125.3–132.3], difference VCI vs. VSI/PRI: *p* = 0.006), the WMI (126.1, 95%-CI [123.9–130.7], difference VSI/PRI vs. WMI: nonsignificant) and the PSI (115.8, 95%-CI [111.9–119.7], difference WMI vs. PSI: *p* < 0.001; Figure 2), in that order. The GC’s mean PSI was nevertheless one SD above the normative value.

For the passive lexicon, the GC’s mean [95%CI] EVIP score (128.7 [125.2–132.2]) was almost two SDs greater than the normative TDC’s score.

### The GC/EVALEO-TDC *p*-ratio for high and low EVALEO scores

**Figure 3:**
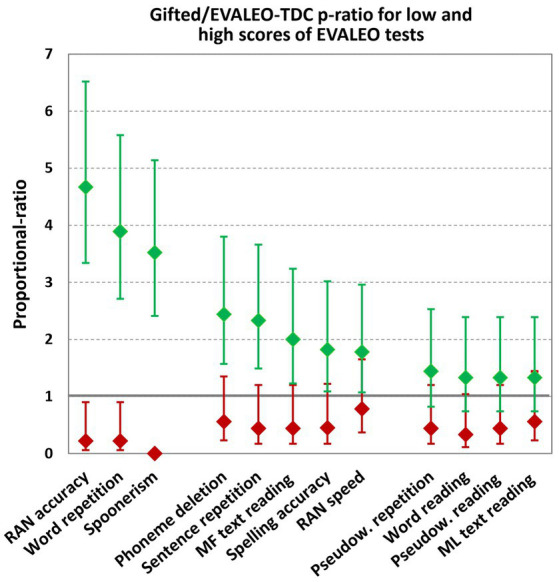
Proportional-ratio (*p*-ratio) of GC vs. EVALEO-TDC norms for scores 1 with 2 (S.12) and scores 6 with 7 (S.67) for the different speech and reading tasks of the EVALEO battery. From left to right, tasks were ordered from the largest to the smallest difference in the GC/EVALEO-TDC *p*-ratio. Vertical bars represent the 95%-CI for low scores (S.12) in red and for high scores (S.67) in green. A 95%-CI *p*-ratio for S.12 of less than one indicates that GC were significantly less likely to score 1 or 2 than EVALEO-TDC, while a 95%-CI *p*-ratio for S.67 greater than 1 indicates that GC were significantly more likely to score 6 or 7 than EVALEO-TDC (95%-CI *p*-ratio were not corrected for multiple comparisons). In example for the RAN accuracy, the GC were 4.7 more likely to achieve S.67 scores than EVALEO-TDC (*p*-ratio = 4.7, 95%-CI between 3.34 and 6.52), while they were 4.5 less likely to achieve S.12 scores than EVALEO-TDC (*p*-ratio = 0.22, 95%-CI between 0.06 and 0.90). MF: meaningful; ML: meaningless; Pseudow., pseudowords.

The GC/EVALEO-TDC *p*-ratio showed that the GC were between 4.7 and 3.5 times more likely than EVALEO-TDC to obtain a score of S.67 for RAN accuracy, word repetition, and the metaphonologic test of spoonerism; the 95%CI ranged from 2.4 to 6.5 (Figure 3). In contrast, the GC were 4.5 times less likely to obtain a score of S.12 than the EVALEO-TDCs; the *p*-ratio ranged from 0 to 0.22, and the highest value of the upper boundary of the 95%CI was 0.90.

For the metaphonologic test of phoneme deletion, sentence repetition, and meaningful text reading, spelling accuracy of writing sentences, and RAN speed, the *p*-ratio for S.67 ranged from 1.8 to 2.4 and the lower boundary of the 95%CI ranged from 1.1 and 3.8. For S.12, the GC/EVALEO-TDC *p*-ratio ranged from 0.44 to 0.78 but the upper boundary of the 95%CI was always greater than 1.

For pseudoword repetition and the ability to read words, pseudowords and meaningless texts, the GC/EVALEO-TDC *p*-ratio for S.67 ranged from 1.33 to 1.44 and the lower boundary of the 95%CI was always below 1. The GC/EVALEO-TDC *p*-ratio for S.12 ranged from 0.33 to 0.56, and the upper boundary of the 95%CI was always above 1.

### Pairwise comparisons of GC and EVALEO-TDC for the EVALEO test results

**Figure 4:**
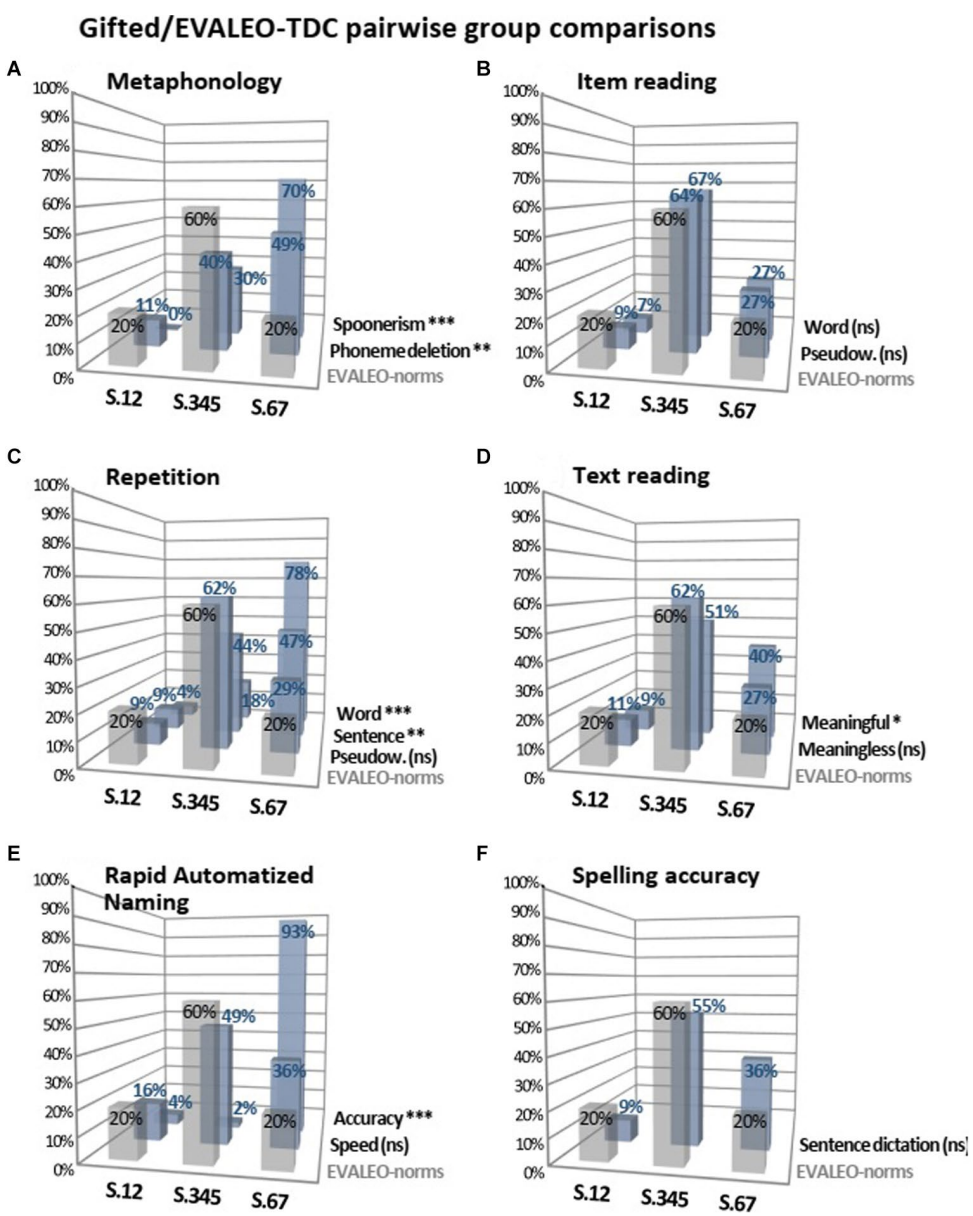
Percentage of GC (blue vertical bars) and EVALEO-TDC norms (grey vertical bars) that obtained S.12, S.345, and S.67 for speech and reading tasks of the EVALEO battery. Groups comparisons were tested with the ki-square and *p*-values were given after Benjamini-Hochberg corrections for multiple comparisons with k = 15 (***, *p* < 0.001; **, *p* < 0.01; *, *p* < 0.05). ns, non significant.

For verbal tests that assessed underlying skills of reading, statistical analysis indicated that GC scored significantly better than EVALEO-TDC for spoonerism and phoneme deletion (*p* < 0.001) and for word and sentence repetition (*p* < 0.001 and *p* = 0.007, respectively). However, the GC’s and EVALEO-TDC’s scores for pseudoword repetition did not differ significantly (Figures 4A,B).

For the RAN, our analysis indicated that GC were significantly better than EVALEO-TDC in terms of accuracy (*p* < 0.001). The GC were better than EVALEO-TDC in terms of reading speed but the difference was not statistically significant (*p* = 0.09; Figure 4C).

For reading words, pseudowords and meaningless texts, the GC and EVALEO-TDC had similar scores (*p* = NS). For reading meaningful texts, the GC’s scores were significantly better than the EVALEO-TDC’s scores (*p* = 0.03; Figures 4D,E).

Lastly, with regard to spelling accuracy, the GC obtained better sentence dictation scores than the EVALEO-TDC but the difference was not statistically significance (*p* = 0.07; Figure 4F).

## Discussion

The results indicated that the GC performed significantly better than the norms in tasks involving automated rapid naming, phoneme deletion, spoonerism, and repetition of words and sentences. Our findings were consistent with those of Van Viersen’s study of the Dutch langage (Van Viersen et al., 2015) and indicated that the GC performed better than TDC in almost all the phonological and metaphonological tests of the components underlying reading skills. According to the SVR model (Sprenger-Charolles and Ziegler, 2019), phonological abilities are involved in the first level of reading processes in general and in the decoding of written words in particular. Previously published studies had demonstrated a strong relationship between metaphonology and working memory (Brady, 1991; Gindri et al., 2007). Thus, the GC’s strong performance in metaphonologic tests might result from their high working memory capacity (see also Aubry et al., 2021). Indeed, the GC’s mean score for the WMI was almost two SDs above the TDC’s mean score.

In the present study, the GC were more efficient than TDC’normative scores in reading meaningful texts. However, in contrast to Van Viersen’s findings, GC were not significantly better than TDC (i) at reading meaningless texts, isolated pseudowords, and complex words, and (ii) with regard to spelling accuracy. The SVR model suggests that reading involves more than just identifying words: it ultimately requires comprehension of the meaning formed by the words identified during the decoding. This last comprehension step involves vocabulary, semantics, and morphosyntactic knowledge, in addition to working memory. Previously published studies have shown strong positive correlations between the passive lexicon and the VCI and even the FSIQ (Hodapp and Gerken, 1999; Caroff et al., 2006). Furthermore, it is known that a rich vocabulary is related to verbal comprehension (Schelstraete, 2012; Haft et al., 2016).

The GC in the present study had a larger passive vocabulary than the TDC’s normative scores, with a EVIP mean score nearly 2 SD higher than the age average. The GC also presented very high semantic knowledge and verbal inference scores; for the latter, the GC’s VCI in the WISC or WPPSI was more than two SDs greater than that of the TDC’s normative scores. Compared with TDC, the GC’s greater semantic and verbal inference abilities allowed them to make much better use of the semantic context of a text, as demonstrated by their excellent ability to read meaningful texts. Our results support the hypothesis whereby decoding speed is linked to comprehension skills (as in the SVR model; Gough and Tunmer, 1986) and also knowledge of the text’s structure (as in the Active View of Reading model; Duke and Cartwright, 2021). In contrast, the GC performing no better than TDC with regard to semantic abilities that cannot be used, i.e., reading meaningless texts. Similarly, there is no semantic context to be leveraged when reading isolated words or pseudowords. Indeed, GC did not outperform TDC’s normative scores when reading meaningless texts, isolated words, and pseudowords. With regard to spelling accuracy, French language rules are complex, often arbitrary, and thus difficult to predict (Fayol and Jaffré, 2008). Once again, it appears that good semantic and verbal inference skills are not linked to spelling accuracy, as shown by GC’s near-normal performance in a sentence dictation.

## Limitations

Firstly, the study population was relatively small. Nevertheless, given that our GC’s cognitive profile for the WISC was similar to those found in other studies, the sample was probably representative of GC in general. Indeed, the GC’s VCI was two SDs higher than the TDC’s VCI. Although the GC’s other indexes were lower in absolute terms, they were significantly better than the TDC’s normative scores. The GC’s lowest score (the PSI) was still one SD greater than the equivalent for the TDC (Liratni and Pry, 2007; Terriot, 2018). The details of the socio-economic status of the GC studied were not known. However, the children in the study were recruited from private schools, in which we can expect a socio-economic status bias quite similar to that of EVALEO population: probable over-representation of executives and higher intellectual professions and under-representation of workers.

We did not assess reading comprehension because this complex cognitive activity is extremely difficult to measure in a standardized test (Bianco, 2019). Nevertheless, many processes involved in reading comprehension are also involved in verbal comprehension (Leloup et al., 2022). In their review of the literature, Snowling and Melby-Lervåg (2016) concluded that a written language comprehension disorder is associated with a verbal language comprehension disorder. However, this was not the case for our GC, who scored highly for the WISC VCI and the EVIP vocabulary test.

The French guidelines on good practice in the assessment, prevention and remediation of written language disorders in children and adults were based on the SVR model (Leloup et al., 2022). Recently, more dynamic reading models (such as the Active View of Reading model) have been built on the SVR model but take account of an overlap between listening comprehension and decoding (Duke and Cartwright, 2021). These models describe more complex, intricate processes.

## Conclusion

The present study covered most reading skills; the results indicated that GC performed better than TDC in tests that assess the underlying skills of reading (i.e., phonology and metaphonology). This difference was probably due to the GC’s highly efficient working memory. The GC were better able to read meaningful texts because they leveraged the semantic context more effectively than TDC did. This was probably due to the GC’s excellent verbal inference abilities and richer vocabulary, compared with TDC. In tests that did not involve underlying reading skills or semantic aspects, GC did not perform any better than TDC; this was observed for reading meaningless texts, isolated words and isolated pseudowords and for spelling accuracy (the rules for which are extremely complex and arbitrary in French). Thus, GC did not have uniformly excellent language and reading skills; some skills were well above-average, and others were within the norm. GC also show heterogeneity in various intellectual domains, which significant differences between scores for verbal reasoning, non-verbal reasoning, working memory, and processing speed.

Some researchers have argued that the threshold for abnormal reading ability should be modified for GC with SLD. For example, Habib (2018) argued that the reading norm for TDC is abnormal threshold for GC with SLD. However, the results of the study argued against this suggestion because our GC (without SLD) and TDC did not differ significantly with regard to certain reading skills of GC. Nevertheless, our results call into question the relevance of the usual reading disorder thresholds for GC. It appears that strict application of the standard abnormal thresholds for GC would result in a large number of false negatives; these children would not receive appropriate care and would not have their disability recognized for school and examination purposes. Our results emphasize the importance of using specific thresholds to assess the intellectual abilities of GC and the need for reference data for reading disorders in this population. Our findings strongly supported Kranz et al.’s conclusion (2024) whereby specific thresholds should be established in this population.

## Data Availability

In accordance with French legislation, the parents were given written information about the study and were free to refuse the inclusion of their child in the study. The datasets cannot be shared for ethical reasons; sharing has not been authorized by the French National Data Protection Commission (Commission nationale de l’informatique et des libertés). Requests to access the datasets should be directed to lesecq.laurent@chu-amiens.fr.
